# Genome-Wide Identification and Comparative Analysis of the 3-Hydroxy-3-methylglutaryl Coenzyme A Reductase (HMGR) Gene Family in *Gossypium*

**DOI:** 10.3390/molecules23020193

**Published:** 2018-01-24

**Authors:** Wei Liu, Zhiqiang Zhang, Wei Li, Wei Zhu, Zhongying Ren, Zhenyu Wang, Lingli Li, Lin Jia, Shuijin Zhu, Zongbin Ma

**Affiliations:** 1 Collaborative Innovation Center of Henan Grain Crops/Agronomy College, Henan Agricultural University, Zhengzhou 450002, China; lw6051@163.com (W.L.); 13598838901@163.com (Z.Z.); zhuwei_2006z@126.com (W.Z.); ndlll@126.com (L.L.); cathylin2012@163.com (L.J.); 2 State Key Laboratory of Cotton Biology/Institute of Cotton Research of Chinese Academy of Agricultural Sciences, Anyang 455000, China; lw8887@163.com (W.L.); renzhongyingcotton@163.com (Z.R.); wangzhenyu89@126.com (Z.W.); 3 Department of Agronomy, Zhejiang University, Hangzhou 310058, China

**Keywords:** *Gossypium*, HMGR, terpene biosynthesis, gene expansion, pseudogene

## Abstract

Terpenes are the largest and most diverse class of secondary metabolites in plants and play a very important role in plant adaptation to environment. 3-Hydroxy-3-methylglutaryl coenzyme A reductase (HMGR) is a rate-limiting enzyme in the process of terpene biosynthesis in the cytosol. Previous study found the *HMGR* genes underwent gene expansion in *Gossypium raimondii*, but the characteristics and evolution of the *HMGR* gene family in *Gossypium* genus are unclear. In this study, genome-wide identification and comparative study of *HMGR* gene family were carried out in three *Gossypium* species with genome sequences, i.e., *G. raimondii*, *Gossypium arboreum*, and *Gossypium hirsutum*. In total, nine, nine and 18 *HMGR* genes were identified in *G. raimondii*, *G. arboreum*, and *G. hirsutum*, respectively. The results indicated that the *HMGR* genes underwent gene expansion and a unique gene cluster containing four *HMGR* genes was found in all the three *Gossypium* species. The phylogenetic analysis suggested that the expansion of *HMGR* genes had occurred in their common ancestor. There was a pseudogene that had a 10-bp deletion resulting in a frameshift mutation and could not be translated into functional proteins in *G. arboreum* and the A-subgenome of *G. hirsutum*. The expression profiles of the two pseudogenes showed that they had tissue-specific expression. Additionally, the expression pattern of the pseudogene in the A-subgenome of *G. hirsutum* was similar to its paralogous gene in the D-subgenome of *G. hirsutum*. Our results provide useful information for understanding cytosolic terpene biosynthesis in *Gossypium* species.

## 1. Introduction

Terpenes are a type of natural compound, which are widely distributed in nature and have diverse structures and functions [1,2]. Thousands of terpenes and derivatives are a good example of metabolic plasticity that is essential to survive in changing environments [3,4]. Additionally, many terpenes are specialized compouds that are rich sources of commercial products widely used as flavors, fragrances and pharmaceuticals by humans [5,6].

In plant cells, terpenes are synthesized by two independent pathways: the mevalonate pathway (MVA pathway) in the cytosol and the 2-*C*-methyl-d-erythritol 4-phosphate pathway (MEP pathway) in the plastid [7,8]. The 3-hydroxy-3-methylglutaryl coenzyme A reductase (HMGR) catalyzes the conversion of 3-hydroxy-3-methylglutaryl coenzyme A (HMG-CoA) to mevalonate (MVA), which is considered to be a rate-limiting enzyme of the MVA pathway and plays a key role in the biosynthesis of plant cytosolic terpenes [9,10]. Currently, *HMGR* genes have been isolated and cloned from many species of plants, such as *Arabidopsis thaliana* [11,12], rice [13], wheat [14], cotton [15], melon [16], medicinal plants *Cymbopogon winterianus*, [17] and *Alisma orientale* [18], and so on. Many experiments have shown that *HMGR* holds an important control point in the MVA pathway and genetic manipulation of *HMGR* indeed increased terpenes content in plants. The *HMGR* gene of *Hevea brasiliensis* was introduced into tobacco by *Agrobacterium* transformation, and then the activity of HMGR in transgenic plants increased by 4–8 times and the total amount of sterols increased by six times [19]. Ginsenosides are glycosylated triterpenoids, and overexpression of the *HMGR* gene in ginseng could significantly increase the amount of ginsenosides [20]. Transgenic spike lavender plants expressing the *Arabidopsis HMGR* gene accumulated more essential oil constituents, which were composed of monoterpenes and sesquiterpenes [21]. Additionally, more and more evidence shows that HMGR is not only critical for normal plant development but also very important in adapting to changing environments. HMGR was negatively regulated by protein phosphatase 2A (PP2A) in *Arabidopsis* plants during development and in response to stress conditions [22]. In *Malus domestica*, the various putative *cis*-acting elements were present in the promoter of *MdHMGR1*, *MdHMGR2* and *MdHMGR4* to response to different hormones, and the expression patterns of *MdHMGR2* and *MdHMGR4* were significantly induced by ethephon (ETH), methyl jasmonate (MeJA), and salicylic acid (SA) [23,24]. In *Origanum vulgare* subsp. *gracile*, the expression of the *HMGR* gene was directly affected by the changing environmental condition and was enhanced under water stress conditions [25].

The *Gossypium* (cotton) genus contains 50 species, of which 45 diploid (2n = 2x = 26) and five tetraploid (2n = 4x = 52) species, and all diploid cotton species are divided into eight genomes, A, B, C, D, E, F, G and K [26]. At present, two diploid cottons, *Gossypium raimondii* (D_5_) [27,28] and *Gossypium arboreum* (A_2_) [29], and the tetraploid cotton, *Gossypium hirsutum* ((AD)_1_) [30,31] had completed whole genome sequencing. *G. raimondii* is a wild species belonging to the D-genome, and *G. arboreum* is a cultivar belonging to the A-genome [28,29]. They diverged from the same progenitor approximately 5–10 million years ago and *G. arboreum* underwent artificial domestication and selection [26,29]. Tetraploid cotton species are considered to be produced by interspecific hybridization between the African ancestor of an A-genome resembling *G. arboreum* and the American ancestor of a D-genome resembling *G. raimondii* approximately 1–2 million years ago [26,30]. *G. hirsutum*, as one of tetraploid species, is domesticated to provide the world’s most natural textile fiber and become a major oilseed crop [30,31]. Additionally, *Gossypium* species serve as an ideal plant for studies of genome evolution and polyploidization [32,33]. *Gossypium* plants are also known to produce a specialized group of terpenes in the cytosol, including gossypol and related sesquiterpenoids, which could be used as phytoalexins in plant defense against pests and pathogens, as well as anticancer agents and male contraceptives in humans [34,35].

In previous studies, only a small portion of *HMGR* genes from cotton have been characterized, and our evolutionary analysis indicated that the *HMGR* genes underwent gene expansion and a unique gene cluster containing four *HMGR* genes was found in the diploid cotton, *G. raimondii* [36]. In order to explore the characteristics of the *HMGR* gene family in *Gossypium* genus, the *HMGR* gene family was identified in *G. raimondii*, *G. arboreum*, and *G. hirsutum* at a genome-wide level, and the phylogenetic relationship, chromosomal localization, gene structure, and protein motifs of *HMGR* genes in the three genomes were comprehensively analyzed in this study.

## 2. Results

### 2.1. Genome-Wide Identification of HMGR Genes in Gossypium

The candidate *HMGR* genes were identified from the *Gossypium* genome using the local blast program with the query sequences of *Arabidopsis HMGR* genes. The obtained sequences were submitted to the Pfam database to confirm the presence of conserved domains (PF00368). Then these sequences were further submitted to the Interpro database and validated to be the *HMGR* gene family membership (IPR004554). Finally, nine, nine and 18 putative *HMGR* genes were identified in *G. raimondii*, *G. arboreum*, and *G. hirsutum*, respectively (Table 1 and Supplementary Materials Table S3). The *HMGR* genes in *G. raimondii* were preferentially named *GrHMGR1* to *GrHMGR9*, according to the published article [36]. Based on the orthologs in *G. raimondii*, the *HMGR* genes in *G. arboreum* were named *GaHMGRs*, with the same number as in *G. raimondii*. The *HMGR* genes in *G. hirsutum* were named *GhHMGRs* corresponding to the orthologs in *G. raimondii* and *G. arboreum*, and the D and A subgenomes were represented by suffixes D and A after each gene names, respectively. The genomic sequences with the upstream and downstream sequences of these gene loci were extracted and the coding sequences of these genes were re-predicted by the gene annotation tool FGENESH [37]. Then all the coding sequences were further manually verified by RT-PCR using gene-specific primers. As a result, 34 gene loci were confirmed to have the complete open reading frame (Supplementary Materials Table S4), while the *G. arboreum GaHMGR1* and *G. hirsutum GhHMGR1A* were pseudogenes with a premature stop codon in their coding sequences. Compared with the initial annotation in genome database, the coding sequences of six *HMGR* genes (*GhHMGR3A*, *GhHMGR4A*, *GhHMGR8A*, *GhHMGR8D*, *GaHMGR2*, and *GaHMGR9*) were modified. Interestingly, the two gene loci (*GhHMGR3A* and *GhHMGR4A*) were originally found in one gene locus in the genome database of *G. hirsutum*, and their coding sequences were re-annotated subsequently. The coding sequences of *GhHMGR8A* and *GhHMGR8D* had a deletion according to the sequencing results. Additionally, the coding sequences of *GaHMGR2* and *GaHMGR9* had an insertion compared with their initial annotations. Overall, the results showed that *G. raimondii* and *G. arboreum* had the same the number of *HMGR* loci (nine loci) and that *G. hirsutum* had twice as many *HMGR* loci as the other two species (18 loci).

One salient feature of the plant HMGR proteins is that they usually have two transmembrane movements across the endoplasmic reticulum membrane, and their catalytic domains are exposed to the cytosol during the process of performing function [38,39]. The transmembrane domains of *Gossypium* HMGR proteins were predicted by the online prediction tool TMHMM Server v. 2.0 (Supplementary Materials Figures S1–S3). The results showed that all the 34 HMGR proteins had two transmembrane domains at the N-terminus, and it was deduced that, as with other plant *HMGR* genes, it was necessary for *Gossypium* HMGR proteins to anchor on the membrane by two transmembrane movements in the catalysis reaction.

### 2.2. Chromosomal Distribution and Phylogenetic Analysis of Gossypium HMGR Genes

In *G. raimondii*, the nine *HMGR* genes were found in five chromosomes: four on chromosome 5, two on chromosome 2, and one on chromosome 8, 12 and 13 each (Supplementary Materials Figure S4). Although the chromosome distribution was found to be little different from the published article [36], there was also an *HMGR* gene cluster containing four genes on *G. raimondii* chromosome 5 (*GrHMGR2*, *GrHMGR3*, *GrHMGR4* and *GrHMGR5*).

Likewise, in *G. arboreum*, the nine *HMGR* genes were found in five chromosomes (Supplementary Materials Figure S5). There was a single *HMGR* gene locus on chromosome 4, 6 and 13 each, and two *HMGR* gene loci on chromosome 7, but one of them was a pseudogene (*GaHMGR1*). Similarly, there was also an *HMGR* gene cluster on *G. arboreum* chromosome 5 (*GaHMGR2*, *GaHMGR3*, *GaHMGR4* and *GaHMGR5*). Simultaneously, the 18 *HMGR* genes were found in *G. hirsutum*: nine for each of the D-subgenome and the A-subgenome (Supplementary Materials Figure S6). There was a pseudogene named as *GhHMGR1A* in the A-subgenome. Additionally, both of the two subgenomes had an *HMGR* gene cluster that contained four closely adjacent genes (*GhHMGR2D*, *GhHMGR3D*, *GhHMGR4D* and *GhHMGR5D* in the *G. hirsutum* D-subgenome, and *GhHMGR2A*, *GhHMGR3A*, *GhHMGR4A* and *GhHMGR5A* in the *G. hirsutum* A-subgenome), which was consistent with the two diploid cottons, *G. raimondii* and *G. arboreum*.

**Figure 1:**
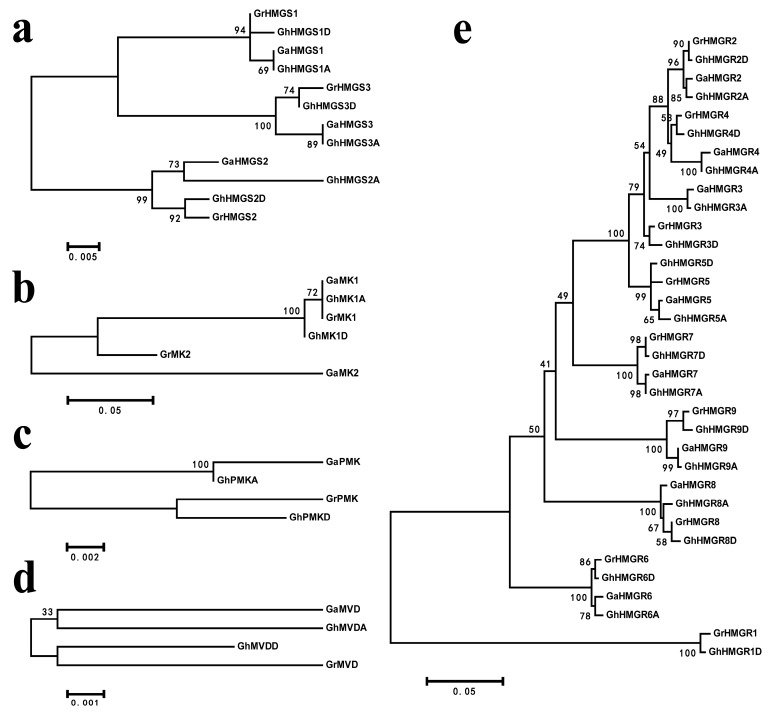
Phylogenetic relationship of HMGS, MK, PMK, MVD and HMGR proteins from *G. raimondii*, *G. arboreum* and *G. hirsutum*. (**a**) HMGS; (**b**) MK; (**c**) PMK; (**d**) MVD; (**e**) HMGR. Numbers at the nodes represent bootstrap support values (1000 replicates). The bars in (**a**–**e**) indicate 0.5%, 5%, 0.2%, 0.1% and 5% sequence divergence, respectively.

The genes encoding other related enzymes in upstream or downstream of HMGR in the MVA pathway were also identified in *Gossypium*. As a result, there were three genes encoding 3-hydroxy-3-methylglutaryl coenzyme A synthase (HMGS), two genes encoding mevalonate kinase (MK), one gene encoding phosphomevalonate kinase (PMK) and one gene encoding mevalonate diphosphate decarboxylase (MVD) in each of *G. raimondii* and *G. arboreum*, and six *HMGS* genes, two *MK* genes, two *PMK* genes and two *MVD* genes in *G. hirsutum* (Supplementary Materials Table S5). Phylogenetic trees based on the protein sequences of these genes in the MVA pathway of *Gossypium* were constructed to investigate the evolutionary relationships (Figure 1).

**Figure 2:**
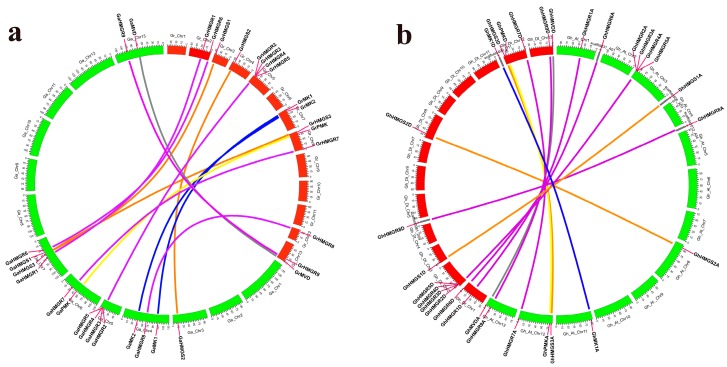
Locations and homologous relationships of the MVA pathway genes in *G. raimondii* and *G. arboreum*, and in the A-subgenome and D-subgenome of *G. hirsutum*. (**a**) Locations and orthologous relationships of the MVA pathway genes in *G. raimondii* and *G. arboreum*; (**b**) Locations and paralogous relationships of the MVA pathway genes in the D-subgenome and the A-subgenome of *G. hirsutum*. The chromosomes of *G. raimondii*, *G. arboreum*, *G. hirsutum* D-subgenome, and *G. hirsutum* A-subgenome are shown with different colors and labeled as Gr, Ga, Gh_Dt and Gh_At, respectively. The putative homologous gene pairs belonging to the *HMGS*, *HMGR*, *MK*, *PMK* and *MVD* gene families are connected by orange, purple, blue, yellow and grey lines, respectively. Several genes are located on the scaffolds that do not determine the exact locations and are placed next to the corresponding chromosomes.

**Figure 3:**
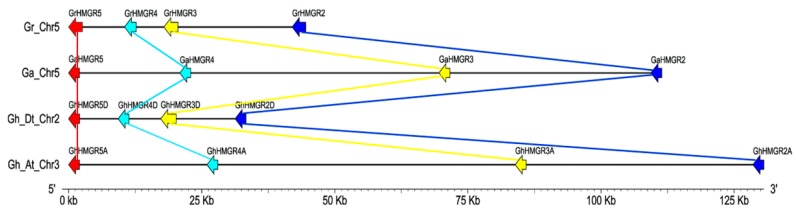
The *HMGR* gene clusters in *G. raimondii*, *G. arboreum* and *G. hirsutum*. The putative homologous gene pairs are displayed by arrows of the same color and connected by lines of the same color. The direction of arrows indicates the direction of transcriptions.

The position of each gene and the homologous gene pairs were displayed intuitively with Circos diagrams (Figure 2). We identified 16 pairs of orthologous genes between *G. raimondii* and *G. arboreum* and 15 pairs of paralogous genes between the D-subgenome and the A-subgenome of *G. hirsutum*. It showed that there were one-to-one relationships between homologous genes of the MVA pathway in the two diploid cottons or in the two subgenomes of the tetraploid cotton. For the three *HMGS* genes and two *MK* genes in *G. raimondii*, they had orthologous genes in *G. arboreum*. In *G. hirsutum*, the three *HMGS* genes and one *MK* gene in the D-subgenome had corresponding paralogs in the A-subgenome. However, the orthologous genes of *GaMK2* and *GrMK2* were not found in *G. hirsutum*, indicating that they might be lost during the formation of *G. hirsutum*. For the *PMK* genes, including *GaPMK*, *GrPMK*, *GhPMKA* and *GhPMKD*, they exhibited the corresponding homologous relationships in *G. raimondii* and *G. arboreum* or the two subgenomes of *G. hirsutum*. Similarly, two *MVD* genes as a paralogous gene pair in *G. hirsutum* also had a corresponding ortholog in *G. raimondii* and *G. arboreum*. Furthermore, we elaborately detected the homologous relationships of *HMGR* genes, two *HMGR*s on chromosome 2, four *HMGR*s in a gene cluster on chromosome 5, three *HMGR*s on chromosome 8, 12 and 13 of *G. raimondii* had one to one orthologous relationships with two *HMGR*s on chromosome 7, four *HMGR*s in a gene cluster on chromosome 5, three *HMGR*s on chromosome 4, 6 and 13 of *G. arboreum*. Additionally, five *HMGR*s on chromosome 1, scaffold31_A01, scaffold1012_A04, 12 and 13, and four *HMGR*s in a gene cluster on chromosome 3 of the A-subgenome had one to one corresponding paralogous relationships with five *HMGR*s on chromosome 1, scaffold3981_D04, 12 and 13, and four *HMGR*s in a gene cluster on chromosome 2 of the D-subgenome of *G. hirsutum*. In general, it indicated that only the *HMGR* gene formed a gene cluster containing four genes and the gene cluster was present in all three cotton genomes (Figure 3).

### 2.3. Gene Structure and Conserved Protein Motifs of Gossypium HMGR Genes

**Figure 4:**
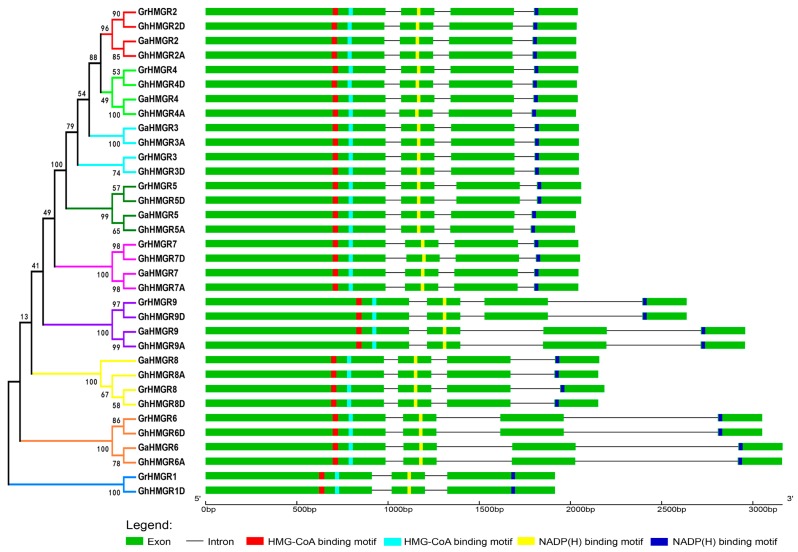
Phylogenetic relationship, gene structure, and conserved motifs of *HMGR* genes from *G. raimondii*, *G. arboreum* and *G. hirsutum*. Exons are represented by green boxes and introns by black lines. The two HMG-CoA binding motifs (EMPVGYVQIP and TTEGCLVA) and two NADP(H) binding motifs (DAMGMNM and GTVGGGT) are represented by red, light blue, yellow and dark blue boxes, respectively.

The gene structure of *Gossypium HMGR* genes was determined (Figure 4). Except for *GrHMGR1* and *GhHMGR1D*, the majority of protein-coding *HMGR* genes in the three *Gossypium* species had the typical gene structure with three introns and four exons, which was the same as the gene structure of most *HMGR* genes in plants [36]. *GrHMGR1* and *GhHMGR1D* lacked the last intron, becoming the structure with two introns and three exons. Most of the *HMGR* genes had almost the same length of exons, only intron length varied greatly. The second and third introns of *GaHMGR6*, *GhHMGR6A*, *GrHMGR6* and *GhHMGR6D* were relatively long. *GaHMGR9*, *GhHMGR9A*, *GrHMGR9* and *GhHMGR9D* had also changed the gene structure. Although they had almost the same exon length as each other, their first exon was longer than other *HMGR* genes. Additionally, *GaHMGR9* and *GhHMGR9A* had a short insertion in the second intron compared with *GrHMGR9* and *GhHMGR9D*. The *HMGR* genes in the gene clusters had almost the same gene structure.

In the catalytic domain of the HMGR proteins, there were four highly conserved motifs: two HMG-CoA binding motifs (EMPVGYVQIP and TTEGCLVA) and two NADP(H) binding motifs (DAMGMNM and GTVGGGT) [36,40,41]. All 34 *HMGR* proteins of the three *Gossypium* species had the four conserved motifs, in which the relative position of these motifs was also conserved and remained constant (Figure 4 and Supplementary Materials Figure S7). Specifically, the first HMG-CoA binding motif (EMPVGYVQIP) was separated from the second HMG-CoA binding motif (TTEGCLVA) by 19 amino acid residues, there was the first NADP(H) binding motif (DAMGMNM) after 88 amino acid residues and the second NADP(H) binding motif (GTVGGGT) was at the C-terminus, separated from the first NADP(H) binding motif by 142 amino acid residues. The sequences of *GaHMGR9*, *GhHMGR9A*, *GrHMGR9* and *GhHMGR9D* at the N-terminus before the first HMG-CoA binding motif were longer than that of other *HMGR* genes, whereas *GrHMGR1* and *GhHMGR1D* were shorter than other genes in this region.

### 2.4. Identification of HMGR Pseudogenes

**Figure 5:**
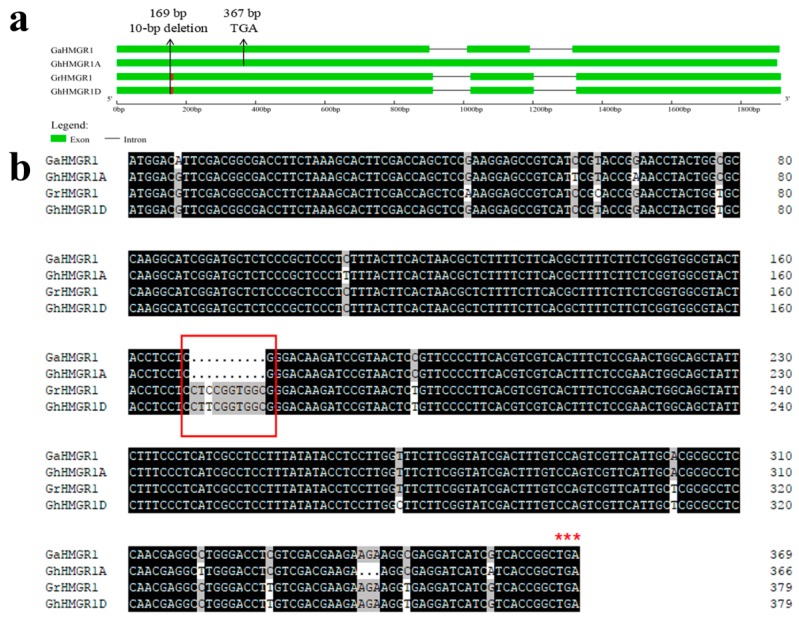
The *HMGR* pseudogenes in *G. arboreum* and *G. hirsutum*. (**a**) The gene structure of *GaHMGR1*, *GhHMGR1A*, *GrHMGR1* and *GhHMGR1D*. Exons are represented by green boxes and introns by black lines. The red boxes in the first exons of *GrHMGR1* and *GhHMGR1D* indicate the 10-bp deletion at the 169-bp position in *GaHMGR1* and *GhHMGR1A*; (**b**) The alignment of predicted coding sequence of *GaHMGR1* and *GhHMGR1A*, and corresponding sequence of *GrHMGR1* and *GhHMGR1D*. The red outlined box indicates the 10-bp deletion of *GaHMGR1* and *GhHMGR1A*. The three red stars indicate the premature stop codon (TGA) at the 367-bp position of *GaHMGR1* and *GhHMGR1A*.

We found a special *HMGR* gene locus *GaHMGR1* in *G. arboreum*. The sequence alignment of *GaHMGR1* and its orthologous gene in *G. raimondii GrHMGR1* showed that their sequences were very similar, and there were only a few nucleotide insertion, deletion and substitution mutations. However, *GaHMGR1* lacked a 10-bp fragment in the first exon region compared with *GrHMGR1*, leading to a premature stop codon (TGA) mutation (Figure 5).

Through cloning and sequencing, it was found that *GaHMGR1* in *G. arboreum* could transcribe the full length RNA sequence similar to the *GrHMGR1*. However, the predicted protein sequence based on the RNA sequence showed that the translation was terminated prematurely due to the advance of the stop codon and the functional protein could not be translated.

Based on the genomic sequence of *GaHMGR1*, an *HMGR* pseudogene in the A-subgenome of *G. hirsutum* was identified by the Blast method and named *GhHMGR1A*. The sequence alignment revealed that its sequence was very similar to *GaHMGR1*. And both of them had the frameshift mutation and premature stop codon (TGA) due to the 10-bp deletion at the same position. These results indicated that *GaHMGR1* and *GhHMGR1A* were really orthologous genes. In particular, we cloned *GhHMGR1A* from *G. hirsutum* (TM-1) using the seedling cDNA as the template, and sequencing results showed that its transcripts contained the whole sequence belonging to exons and introns in its protein-coding counterpart. In order to eliminate the effects of DNA contamination and alternative splicing and further confirm the result, we cloned the pseudogene using the cDNAs of roots, stems, cotyledons, leaves, and petals of TM-1, and at least four clones for the pseudogene from each of the materials were picked randomly and sequenced. Finally, the results indicated that the transcripts were consistent in all materials. Then in order to study the distribution of the *HMGR* pseudogene in *Gossypium*, the pseudogene was identified in several *Gossypium* species, including wild species of the D-genome, wild species and cultivars of A-genome, and wild species, semi-domesticated species and cultivars of the AD-genome. We collected leaves of these species and used their cDNAs to clone the pseudogene with gene-specific primers, and then four clones of each material were picked randomly for sequencing. It was found that all the A-genome species and tetraploid species (AD-genome) we used had the *HMGR* pseudogene (Table 2). Therefore, it could be deduced that the pseudogene was derived from an ancient A-genome species and transferred to the A-subgenome of the tetraploid species during the *Gossypium* evolution.

**Figure 6:**
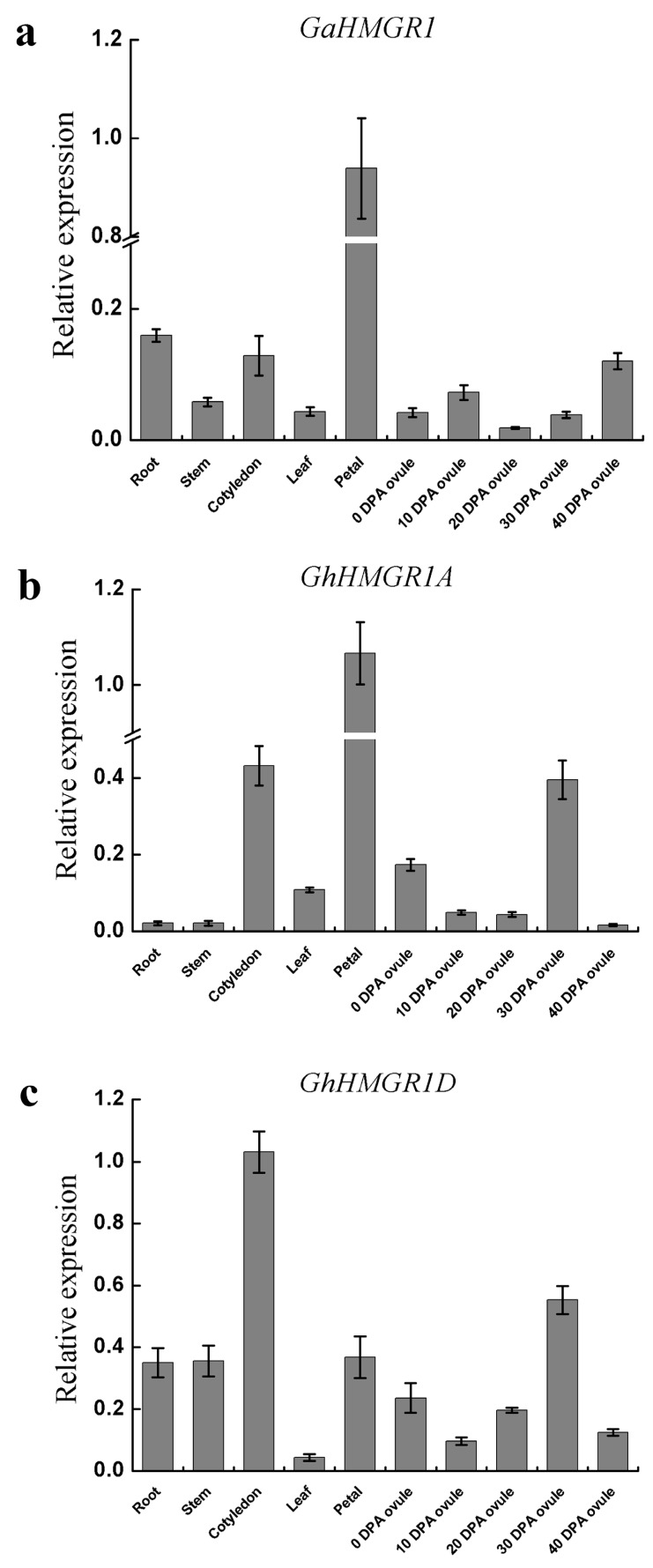
Expression patterns of *GaHMGR1*, *GhHMGR1A* and *GhHMGR1D* in different tissues. (**a**) *GaHMGR1*; (**b**) *GhHMGR1A*; (**c**) *GhHMGR1D*.

Expression patterns of the two *HMGR* pseudogenes (*GaHMGR1* and *GhHMGR1A*) and the homologous gene of *GhHMGR1A* in the D-subgenome of *G. hirsutum* (*GhHMGR1D*) were analyzed by qRT-PCR in roots, stems, cotyledons, leaves, petals, and ovules collected at 0 DPA, 10 DPA, 20 DPA, 30 DPA and 40 DPA of *G. arboreum* and *G. hirsutum* (Figure 6). The results showed that all the three genes displayed tissue-specific expression patterns. Both of the pseudogenes had the highest expression level in petals. In addition, *GaHMGR1* had relatively high expression in roots, cotyledons, ovules at 40 DPA, and had the lowest expression in ovules at 20 DPA. *GhHMGR1A* was highly expressed in cotyledons and ovules at 30 DPA, and lowly expressed in roots, stems and ovules at 40 DPA. Moreover, *GhHMGR1D* as a protein-coding *HMGR* gene, which also was the paralog of the pseudogene *GhHMGR1A* in *G. hirsutum*, was expressed at a high level in roots, stems, cotyledons, petals and ovules at 30 DPA. Although *GhHMGR1D* had the highest expression level in cotyledons, its expression pattern was roughly similar to that of the pseudogene *GhHMGR1A*.

## 3. Discussion

It has been suggested that *HMGR* is a multigene family in cotton and there are seven to nine members in the tetraploid cotton according to the Southern blot technique [15,42]. Our previous studies have shown that there were nine *HMGR* genes in *G. raimondii* and the number of *HMGR* genes was significantly expanded compared with other plants [36]. In this study, another version of *G. raimondii* genome data [27] was used to further confirm that nine *HMGR* gene loci were included in *G. raimondii*. Unexpectedly, the chromosome distribution was little different. In the published study, *GrHMGR6* was located on chromosome 7, but in this study it was on chromosome 2. In addition, the order of four genes in the *HMGR* gene cluster was exactly the opposite. The *G. raimondii* genome used in the previous study [36] was sequenced on the Illumina HiSeq 2000 platform at the BGI-Shenzhen and was assembled using the SOAPdenovo with a *K*-mer of 41 and SSPACE software [28], while the *G. raimondii* genome used in this study was sequenced on the Applied Biosystems 3730xl, Roche 454 XLR and Illumina Genome Analyzer (GA)IIx machines at the U.S. Department of Energy Joint Genome Institute and was assembled using the modified version of Arachne v.20071016 with specific parameters [27]. Considering that the two versions of *G. raimondii* genome data were independent sequenced and assembled, there might be numerous different assembling which might result in the difference of chromosomal distribution of *HMGR* genes. Furthermore, nine and 18 *HMGR* genes were identified in *G. arboreum* and *G. hirsutum*, respectively, using the recently published genome database [29,31]. Phylogenetic analysis showed that the nine *HMGR* genes in *G. arboreum* had one-to-one orthologous relationships with the nine *HMGR* genes in *G. raimondii*, which indicated that these *HMGR* genes were distributed to *G. raimondii* and *G. arboreum* with speciation from the common ancestor of them, and the number of *HMGR* genes has expanded in the ancestral species. The number of *HMGR* genes in *G. hirsutum* was just twice than that of *G. raimondii* and *G. arboreum*, indicating that all the *HMGR* genes were retained in the process of polyploidization.

Gene duplication, including segmental duplication and tandem duplication, has been recognized as the main mechanisms which contributed to expansion of gene families [43,44]. In *Glycine max* and *Populus trichocarpa*, the segmental duplication was the main reason for the expansion of *MYB* and *WRKY* gene families, but there were also some clusters resulting from the tandem duplication [45,46,47,48]. *HMGR* genes were generally distributed on chromosomes dispersedly in other plants [36]. However, there was a gene cluster containing four closely adjacent *HMGR* genes on the chromosome 5 of *G. raimondii* and *G. arboreum*. There was also a gene cluster on the chromosome 3 of the A-subgenome and chromosome 2 of the D-subgenome in *G. hirsutum*. The sequences of four *HMGR* genes in each gene cluster were very similar. It was speculated that the *HMGR* genes had underwent tandem duplication in the common ancestor of *G. raimondii* and *G. arboreum*, leading to the emergence of a *HMGR* gene cluster. Previous studies have shown that segmental duplication and tandem duplication play similar roles in the expansion of the *HMGR* gene family in *G. raimondii* [36]. In this study, it was found that there were nine corresponding *HMGR* gene loci in *G. arboreum*, which further indicated that this segmental duplication and tandem duplication had occurred in the common ancestor of *G. raimondii* and *G. arboreum*, resulting in the expansion of *HMGR* genes. Genomic evolution analysis showed that a whole genome duplication event was uniquely occurred for *Gossypium* after speciation from its closely related species, *Theobroma cacao* [29], which supported the inference that segmental duplication was one of the causes of *HMGR* gene expansion.

In this study, several related genes of the MVA pathway were identified in *G. raimondii*, *G. arboreum* and *G. hirsutum*. There were three *HMGS* genes, nine *HMGR* genes, two *MK* genes, one *PMK* gene, and one *MVD* gene in each of *G. raimondii* and *G. arboreum*, and six *HMGS* genes, 18 *HMGR* genes, two *MK* genes, two *PMK* genes, and two *MVD* genes in *G. hirsutum*. In the model plant *Arabidopsis*, there were one *HMGS* gene, two *HMGR* genes, one *MK* gene, one *PMK* gene and two *MVD* genes [49]. Compared with *Arabidopsis*, the number of *HMGS*, *HMGR* and *MK* gene loci was more in *G. raimondii* and *G. arboreum*, the number of *PMK* loci was the same as that in *Arabidopsis*, and the number of *MVD* loci was one less. In general, only the number of *HMGR* genes in *Gossypium* species was most significantly expanded in the MVA pathway, and there was a unique gene cluster that might have resulted from tandem duplication. *Gossypium* species synthesize gossypol and related sesquiterpenoids uniquely by the MVA pathway in the cytosol and accumulate in roots and pigment glands of aerial tissues, to resist the invasion of pests and pathogens [35,50,51]. The proteins encoded by *HMGR* genes are a rate-limiting enzyme of the MVA pathway and are important regulatory sites for the biosynthesis of terpenes in the cytosol [9,10]. Therefore, it could be speculated that this increase in the number of *HMGR* genes in *Gossypium* species might be likely related to the biosynthesis of more terpenes including gossypol in the cytosol during their growth and development. In addition, this study found that *HMGR* gene expansion and a unique *HMGR* gene cluster were present in the three *Gossypium* species, and the four genes within the *HMGR* gene cluster had almost the same gene and protein structure, which indicated that the gene cluster was quite conserved in the evolutionary process.

After the number of genes was expanded, functional differentiation has three fates: pseudogenization, loss of gene function; neo-functionalization, access to new gene function; sub-functionalization, both of the two copies retain the function of ancestral gene [52]. *GrHMGR1* of *G. raimondii* and its orthologous gene in the D-subgenome of *G. hirsutum*, *GhHMGR1D*, were lacking the third intron compared with other *HMGR* genes. Moreover, its orthologous gene in *G. arboreum*, *GaHMGR1*, and the orthologous gene of *GaHMGR1* in the A-subgenome of *G. hirsutum*, *GhHMGR1A*, had a 10-bp deletion at the same position, resulting in a frameshift mutation, and could not be translated into functional proteins. These results suggested that one functional member after expansion of *HMGR* genes in the common ancestor of *G. raimondii* and *G. arboreum*, might be differentiated by losing the third intron, then it became a pseudogene by losing the 10-bp fragment in the first exon in the ancestor of *G. arboreum* after the speciation of *G. raimondii* and *G. arboreum*. The pseudogene was identified in all the A-genome and AD-genome species collected in this study. Thus, it suggested that the pseudogene might be transferred from wild species to cultivars of the A-genome during process of domestication. Then, during the tetraploid formation by interspecific hybridization between the A-genome and D-genome, the pseudogene was transferred from the A-genome to the A-subgenome.

Previous study has found an *HMGR* pseudogene named *ψhmg5* in *G. hirsutum* and its transcript was detected in cotton embryos [53]. However, because of the lack of genome data of other *Gossypium* species, they were not sure whether the pseudogene arose before or after the polyploidization. Through sequence alignment, we found that the pseudogene identified in the study was the same as the one in the previous study [53]. However, more deeply, we provided a possible mechanism for the formation of the pseudogene by comparative genome analysis. After the expansion of *HMGR* gene in the progenitor of *Gossypium* species, one functional gene might become a new gene with significantly different gene structure attributed to selective excision of the third intron, and led to a pseudogene through a 10-bp deletion in the first exon after a series of evolutionary processes in the A-genome, then transferred to the A-subgenome with polyploidization. In addition, transcripts of the identified pseudogene in *G. hirsutum* contained the whole sequence belonging to exons and introns in its protein-coding counterpart and could be detected in all materials we had collected using the qRT-PCR method.

Pseudogenes do not code for protein, so they have long been labeled as “junk” DNA [54]. However, recent results demonstrated that some pseudogenes could influence their parent genes through their transcripts, including negative regulation and positive regulation [55,56]. For example, the transcripts of pseudogene can produce endogenous siRNAs and then silence the expression of parent gene by RNA interference. In soybean, the inhibition of seed coat pigmentation induced by the *I* gene results from posttranscriptional gene silencing (PTGS) of chalcone synthase (*CHS*) genes and leads to a uniform yellow color of mature harvested seeds [57]. *GmIRCHS* (*Glycine max* inverted-repeat *CHS* pseudogene) was identified as a candidate for the *I* gene [58]. The siRNAs derived from *GmIRCHS* cleaved the mRNA of all *CHS* genes to inhibit their function, and occurred in the seed coat, specifically [59]. Additionally, the pseudogene transcripts can positively regulate its homologous gene by competitively binding miRNAs. For example, *PTEN* is a tumor suppressor gene and maintaining *PTEN* protein levels can inhibit tumorigenesis. Its pseudogene *PTENP1* is highly similar to the homologous coding gene *PTEN* at the 3′ untranslated region (UTR), which can bind miRNAs and reduce the cell concentration of miRNAs, leading to *PTEN* escape from miRNAs repression regulation [60]. In our study, the *HMGR* pseudogenes could still be detected after a long-term evolution, and the pseudogene *GhHMGR1A* showed tissue-specific expression and had the similar expression pattern with its paralogous gene *GhHMGR1D* in the other subgenome. Therefore, it suggested that the pseudogenes might have a potential role in regulation of other *HMGR* genes. Additionally, both of the pseudogenes had the highest expression level in petals. Many plants, such as snapdragon [61], *Hedychium coronarium* [62], and kiwifruit (*Actinidia deliciosa*) [63], can synthesize and emit lots of terpenes in their petals. It could be speculated that the high expression level of the two pseudogenes in petals might be related to the large demand for the precursors for terpene biosynthesis in petals of *G. arboreum* and *G. hirsutum*. Of course, the hypothesis requires further experimental evidence.

## 4. Materials and Methods

### 4.1. Identification of Genes in the MVA Pathway in Gossypium

The genome data of *G. raimondii* [27], *G. arboreum* [29] and *G. hirsutum* [31] were downloaded from the CottonGen database (https://www.cottongen.org/). Then, the local blast database was established for these genome data. The protein sequences of *Arabidopsis thaliana* genes in the MVA pathway were collected from the TAIR database (http://www.arabidopsis.org) [49,64]. BlastP and tBlastN programs were performed against the *Gossypium* genome local databases using the *Arabidopsis* protein sequences as queries with default parameters. All candidates were verified using the Pfam database [65] and InterPro database [66] to identify the members of gene family in the MVA pathway.

### 4.2. Sequence Alignment and Phylogenetic Tree Construction

Multiple sequence alignments were generated using ClustalX software (Version 2.1, Conway Institute UCD, Dublin, Ireland) [67] for the protein sequences with default parameters. Based on the result of multiple sequence alignment, phylogenetic trees were generated using the maximum likelihood method in MEGA software (Version 5.2, Biodesign Institute, Tempe, AZ, USA) [68], using the bootstrap method to assess the reliability with 1000 replicates.

### 4.3. Chromosomal Mapping, Protein Motif, and Gene Structure Analysis

The physical location data of identified genes were retrieved from the *Gossypium* genomes. MapInspect software (http://mapinspect.software.informer.com/) [69] and Circos software (Version 0.67, www.circos.ca) [70] were used to generate chromosomal distribution images for these genes in *G. raimondii*, *G. arboreum* and *G. hirsutum* according to their starting positions on chromosomes. The gene exon/intron structure was drawn using the Gene Structure Display Server (GSDS, http://gsds.cbi.pku.edu.cn/) online tool [71] by comparing the coding sequence (CDS) of each gene with its genomic sequence.

### 4.4. Cotton Plant Growth and Sample Collection

The seeds of *G. arboreum* cv. Shixiya 1, *G. hirsutum* cv. TM-1 and other species used in this study were supplied by Institute of Cotton Research, Chinese Academy of Agricultural Sciences (CAAS, Anyang, China). Whole seedlings, roots, stems, cotyledons and leaves were collected from two-week-old seedlings grown in a greenhouse. Petals were collected from plants on the day of flowering, and ovules were collected at 0, 10, 20, 30 and 40 days post anthesis (DPA). All samples were quick-frozen in liquid nitrogen and stored at −80 °C.

### 4.5. RNA Isolation and cDNA Synthesis

Total RNA was isolated from each sample using the RNA Extraction Kit (TIANGEN, Beijing, China). The RNA concentration was measured using a NanoDrop2000 microvolume spectrophotometer (NanoDrop Technologies, Wilmington, DE, USA) and the integrity of RNA was analyzed on 1% agarose gels. One microgram of total RNA was used for first strand cDNA synthesis using PrimeScript™ 1st Strand cDNA Synthesis Kit (TaKaRa, Dalian, China).

### 4.6. Reverse Transcription PCR (RT-PCR) and Quantitative Real-Time RT-PCR (qRT-PCR)

The gene-specific primers were designed based on the nucleotide sequences by Oligo software (Version 7.60, Molecular Biology Insights, Cascade, CO, USA) and synthesized by Suzhou GENEWIZ (Supplementary Materials Tables S1 and S2). The RT-PCR was carried out as follows: 94 °C for 5 min; followed by 35 cycles of 94 °C for 30 s, 60 °C for 30 s, and 72 °C for 1 min 30 s; then 72 °C for 10 min. The amplified fragments were purified with the MiniBEST Agarose Gel DNA Extraction Kit (TaKaRa, Dalian, China), cloned into the pMD18-Tvector (TaKaRa, Dalian, China) and verified by sequencing. The qRT-PCR was performed using a LightCycler480 system (Roche, Basel, Switzerland) with SYBR^®^ Premix Ex Taq™ (TaKaRa, Dalian, China) and the cotton *UBQ7* gene was used as an internal control. The amplification parameters were as follows: stage 1: 95 °C, 5 min; stage 2: 40 cycles of 95 °C for 10 s, 60 °C for 10 s, 72 °C for 10 s; stage 3: extension at 72 °C for 10 min. Three biological replicates were used for each sample and the results were analyzed using the 2^−ΔΔ^*^C^*^T^ method [72].

## 5. Conclusions

We performed a genome-wide identification of the *HMGR* gene family in *Gossypium* and analyzed their structure, conserved motif, and evolution. The results revealed that the *HMGR* genes were obviously expanded in the common ancestor of *Gossypium* mainly by segmental duplication and tandem duplication, and a gene cluster containing four closely adjacent genes was highly conserved during evolution. There was a pseudogene in *G. arboreum* and the A-subgenome of *G. hirsutum*, and they displayed tissue-specific expression patterns. This study is the first to characterize the *HMGR* gene family in *Gossypium* species and lays an important foundation for further study of cytosolic terpene biosynthesis in cotton.

## Figures and Tables

**Table 1:** The information of *HMGR* genes in *G. hirsutum*.

Gene Name	Gene Locus	Chromosome	Location	Strand	Protein Length	Mw(kDa) ^a^	pI ^a^
*GhHMGR1A* ^c^	*HMGR* pseudogene	A01	39188679-39190582	+	_	_	_
*GhHMGR2A*	Gh_A03G1497	A03	95297747-95299778	−	582	62.33	6.24
*GhHMGR3A* ^b^	Gh_A03G1496_1	A03	95253091-95255136	−	585	62.68	6.14
*GhHMGR4A* ^b^	Gh_A03G1496_2	A03	95195182-95197211	−	585	62.67	6.00
*GhHMGR5A*	Gh_A03G1495	A03	95169197-95171221	−	585	62.71	6.25
*GhHMGR6A*	Gh_A01G2017	scaffold31_A01	38993-42152	+	585	62.82	6.20
*GhHMGR7A*	Gh_A12G0103	A12	1425978-1428020	−	585	62.62	6.24
*GhHMGR8A* ^b^	Gh_A04G1424	scaffold1012_A04	184892-187043	−	581	62.26	6.69
*GhHMGR9A*	Gh_A13G0557	A13	13047920-13050876	−	628	67.60	6.26
*GhHMGR1D*	Gh_D01G1158	D01	25640487-25642401	+	560	60.40	6.53
*GhHMGR2D*	Gh_D02G1965	D02	63580366-63582399	−	582	62.33	6.43
*GhHMGR3D*	Gh_D02G1964	D02	63566380-63569291	−	585	62.54	6.00
*GhHMGR4D*	Gh_D02G1963	D02	63558389-63560423	−	583	62.40	6.17
*GhHMGR5D*	Gh_D02G1962	D02	63549091-63551149	−	585	62.78	6.25
*GhHMGR6D*	Gh_D01G0134	D01	984873-987923	+	585	62.79	5.83
*GhHMGR7D*	Gh_D12G0115	D12	1451036-1453088	−	585	62.44	6.43
*GhHMGR8D* ^b^	Gh_D04G2012	scaffold3981_D04	28507-30658	+	581	62.26	6.49
*GhHMGR9D*	Gh_D13G0573	D13	7846186-7848822	+	628	67.46	6.50

^a^ The theoretical Mw (molecular weight) and pI (isoelectric point) of the full-length protein are predicted by ProtParam tool (http://web.expasy.org/protparam/). ^b^ The coding sequences of genes are re-annotated. ^c^ *GhHMGR1A* is a pseudogene identified in this study.

**Table 2:** Distribution of the *HMGR* pseudogene gene in *Gossypium*.

Species	Type	Genomic Group	*HMGR* Pseudogene
*G. raimondii*	wild species	D_5_	Non-existence
*G. herbaceum* race. *africanum*	wild species	A_1_	Existence
*G. herbaceum* cv. Jinta	cultivar	A_1_	Existence
*G. arboreum* cv. Shixiya1	cultivar	A_2_	Existence
*G. darwinii*	wild species	(AD)_5_	Existence
*G. mustelinum*	wild species	(AD)_4_	Existence
*G. hirsutum* race. *latifolium*	semi-domesticated species	(AD)_1_	Existence
*G. hirsutum* cv. TM-1	cultivar	(AD)_1_	Existence
*G. hirsutum* cv. CCRI41	cultivar	(AD)_1_	Existence
*G. barbadense* cv. Xinhai21	cultivar	(AD)_2_	Existence

## Supplementary Materials

The following are available online. Table S1. Primers for reverse transcription PCR. Table S2. Primers for quantitative real-time PCR. Table S3. The information of *HMGR* genes in *G. raimondii* and *G. arboreum*. Table S4. The coding sequences of *HMGR* genes in *G. raimondii*, *G. arboreum* and *G. hirsutum*. Table S5. The information of *HMGS*, *MK*, *PMK* and *MVD* genes in *G. raimondii*, *G. arboreum* and *G. hirsutum*. Figure S1. Predicted transmembrane domain for *G. raimondii* HMGR proteins. Figure S2. Predicted transmembrane domain for *G. arboreum* HMGR proteins. Figure S3. Predicted transmembrane domain for *G. hirsutum* HMGR proteins. Figure S4. Chromosomal distributions of *HMGR* genes in *G. raimondii*. Figure S5. Chromosomal distributions of *HMGR* genes in *G. arboreum*. Figure S6. Chromosomal distributions of *HMGR* genes in *G. hirsutum*. Figure S7. Phylogenetic relationship and distribution of conserved motifs in HMGR proteins from *G. raimondii*, *G. arboreum* and *G. hirsutum*.
