# Supplementary figures and images for: Genome-Wide Identification and Comparative Analysis of the 3-Hydroxy-3-methylglutaryl Coenzyme A Reductase (HMGR) Gene Family in Gossypium
Source: Molecules. 2018 Jan 24;23(2):193. doi: 10.3390/molecules23020193 (PMC6017885; doi:10.3390/molecules23020193)

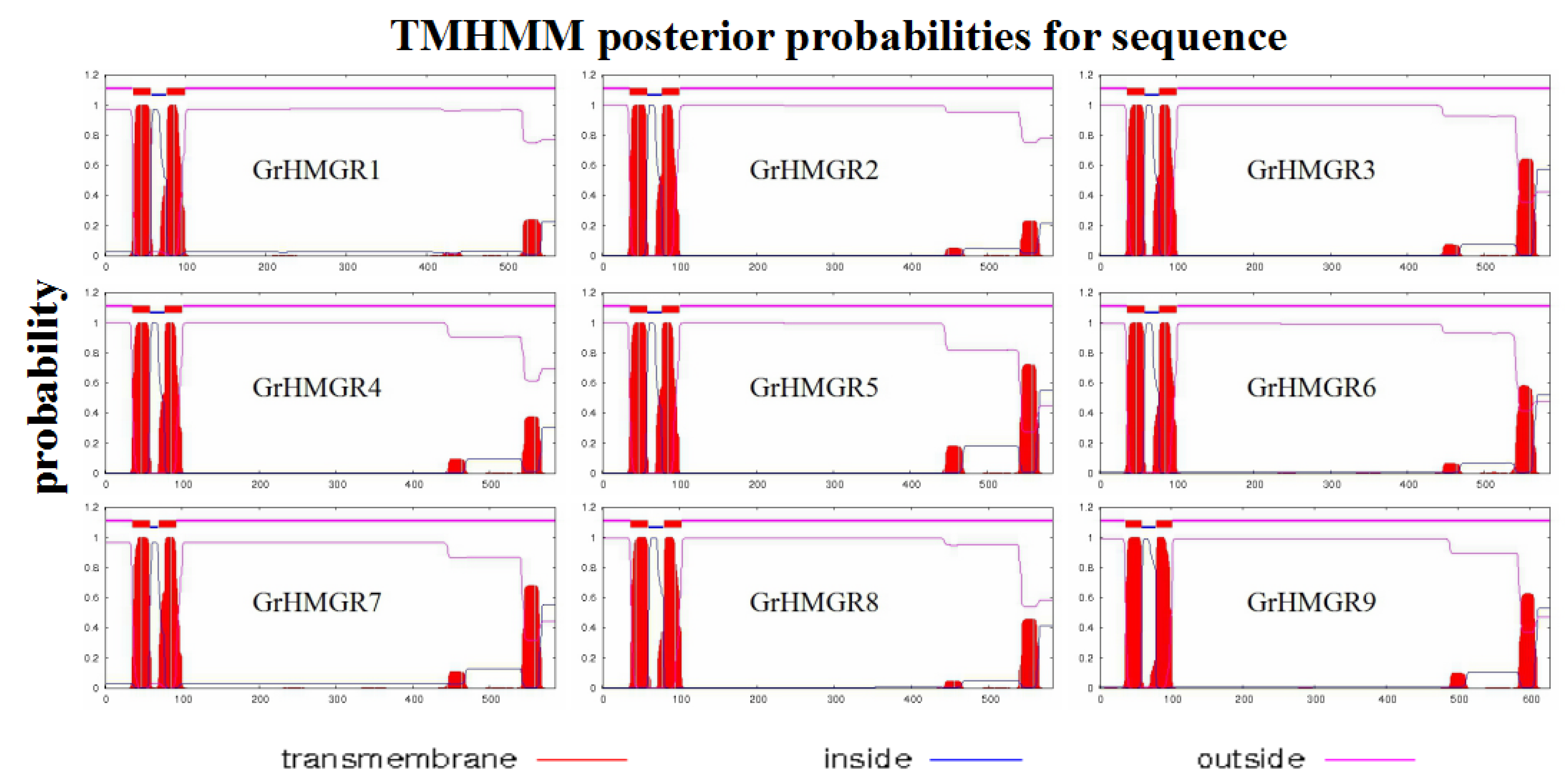

Supplement: Supplementary file 1 [file molecules-23-00193-s001.zip › Supplementary Files/Figure S1.tif]

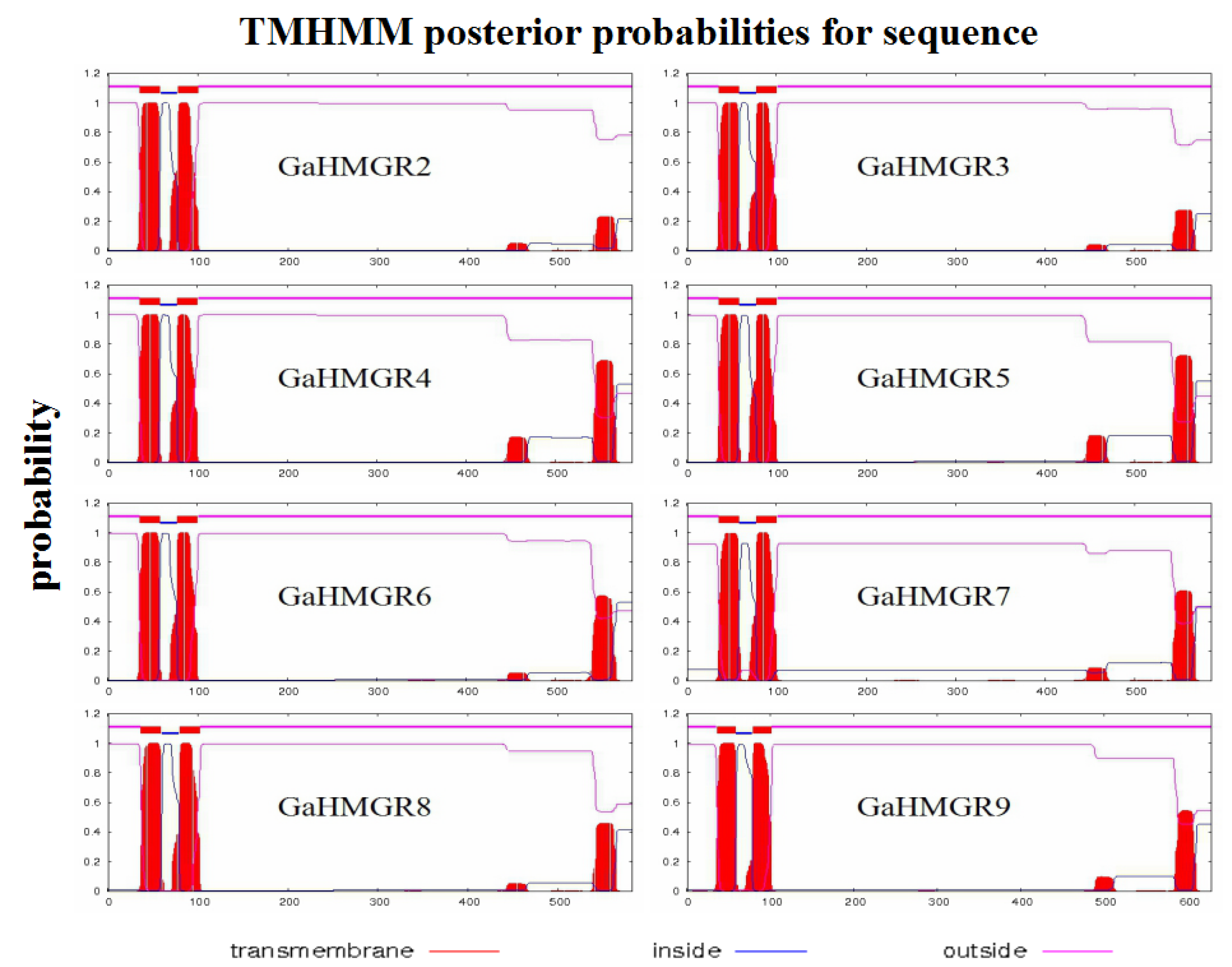

Supplement: Supplementary file 1 [file molecules-23-00193-s001.zip › Supplementary Files/Figure S2.tif]

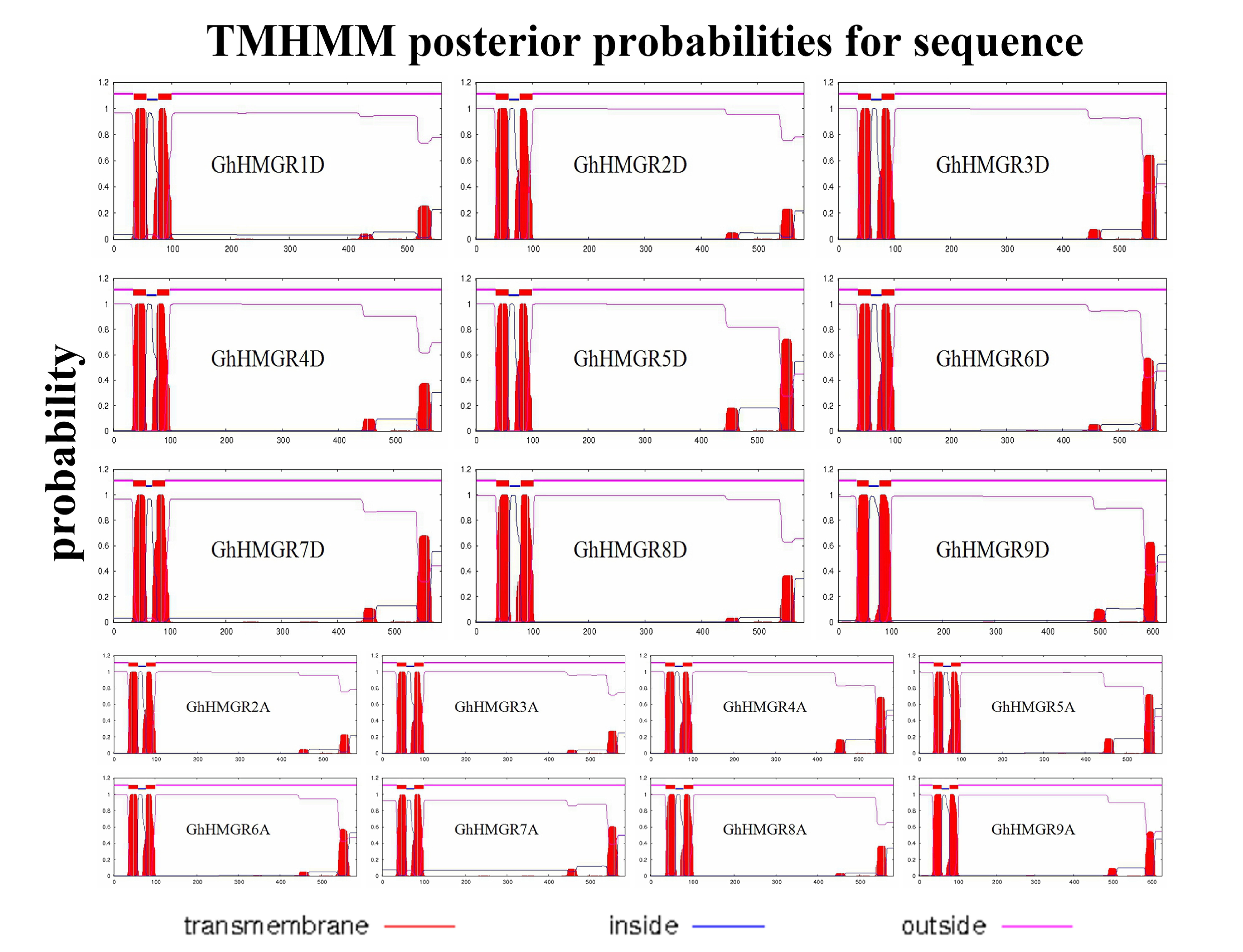

Supplement: Supplementary file 1 [file molecules-23-00193-s001.zip › Supplementary Files/Figure S3.tif]

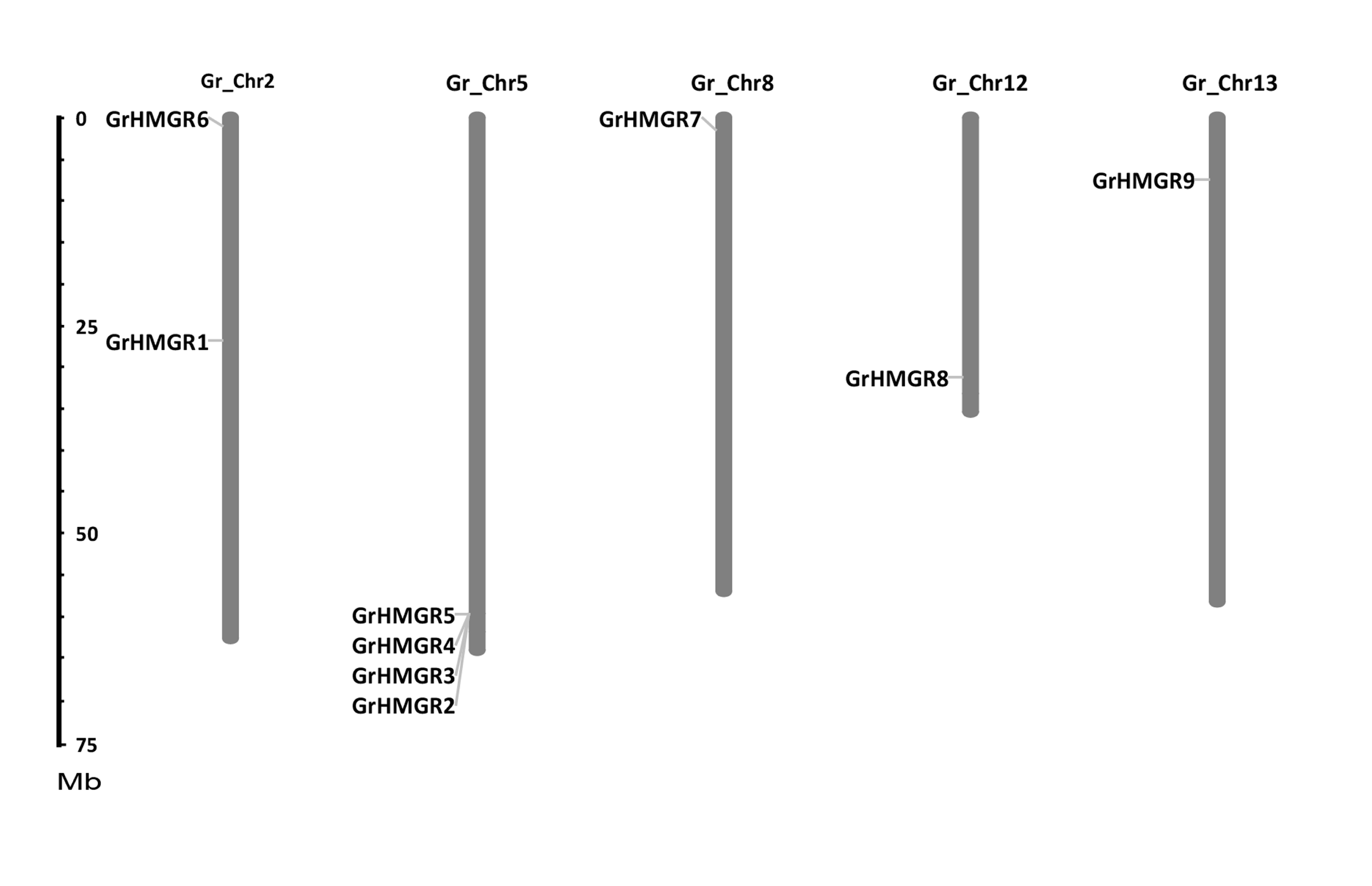

Supplement: Supplementary file 1 [file molecules-23-00193-s001.zip › Supplementary Files/Figure S4.tif]

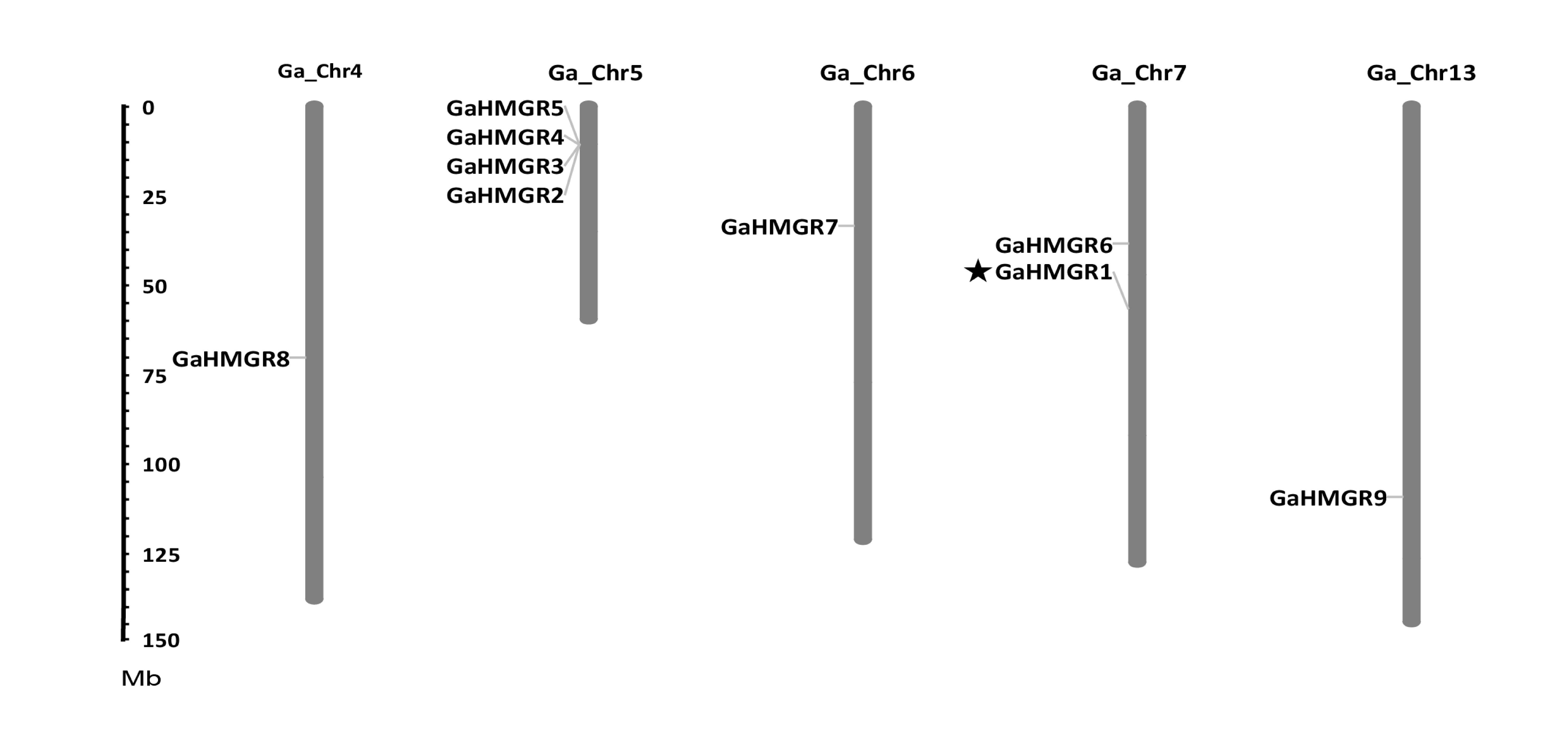

Supplement: Supplementary file 1 [file molecules-23-00193-s001.zip › Supplementary Files/Figure S5.tif]

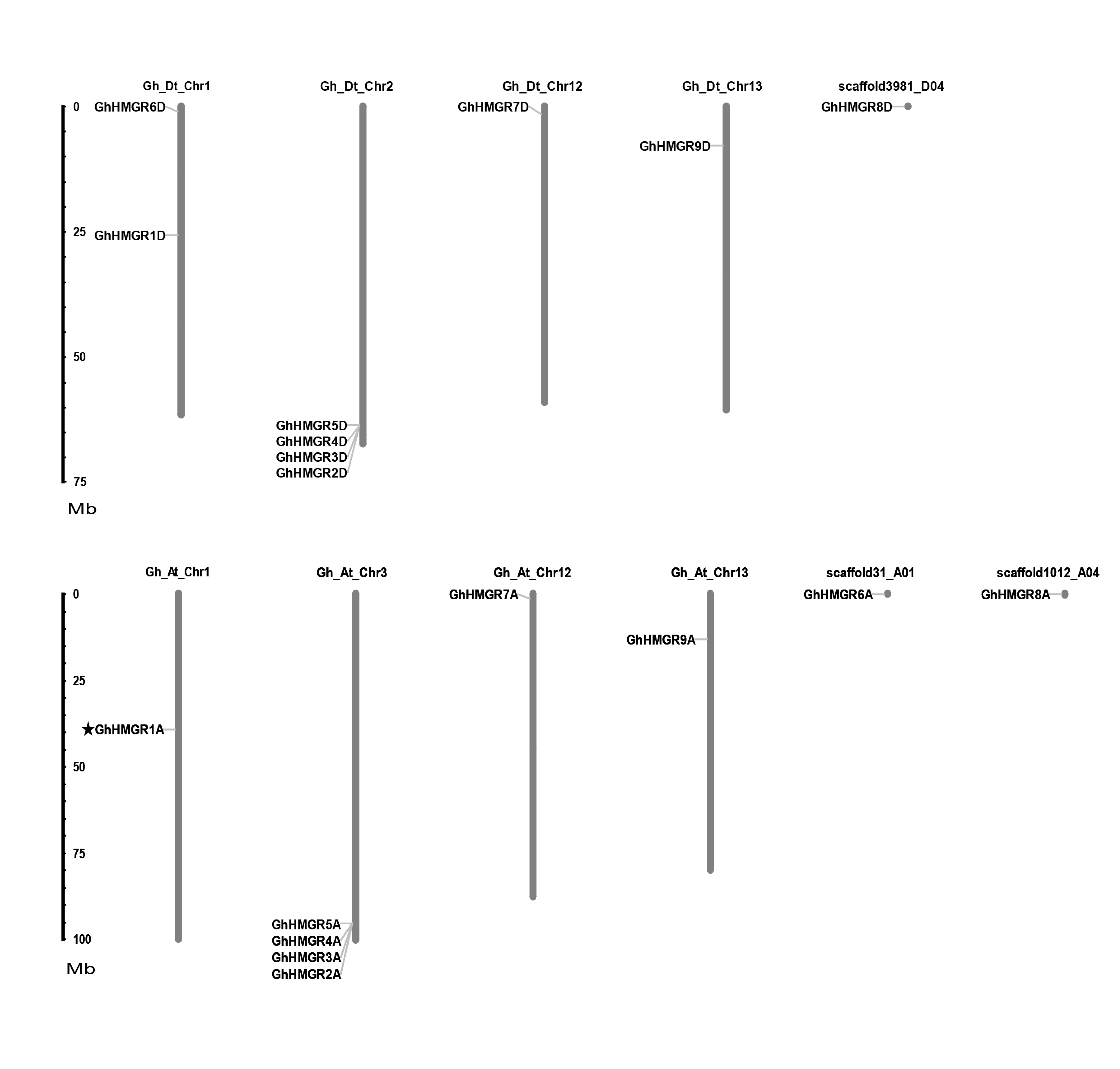

Supplement: Supplementary file 1 [file molecules-23-00193-s001.zip › Supplementary Files/Figure S6.tif]

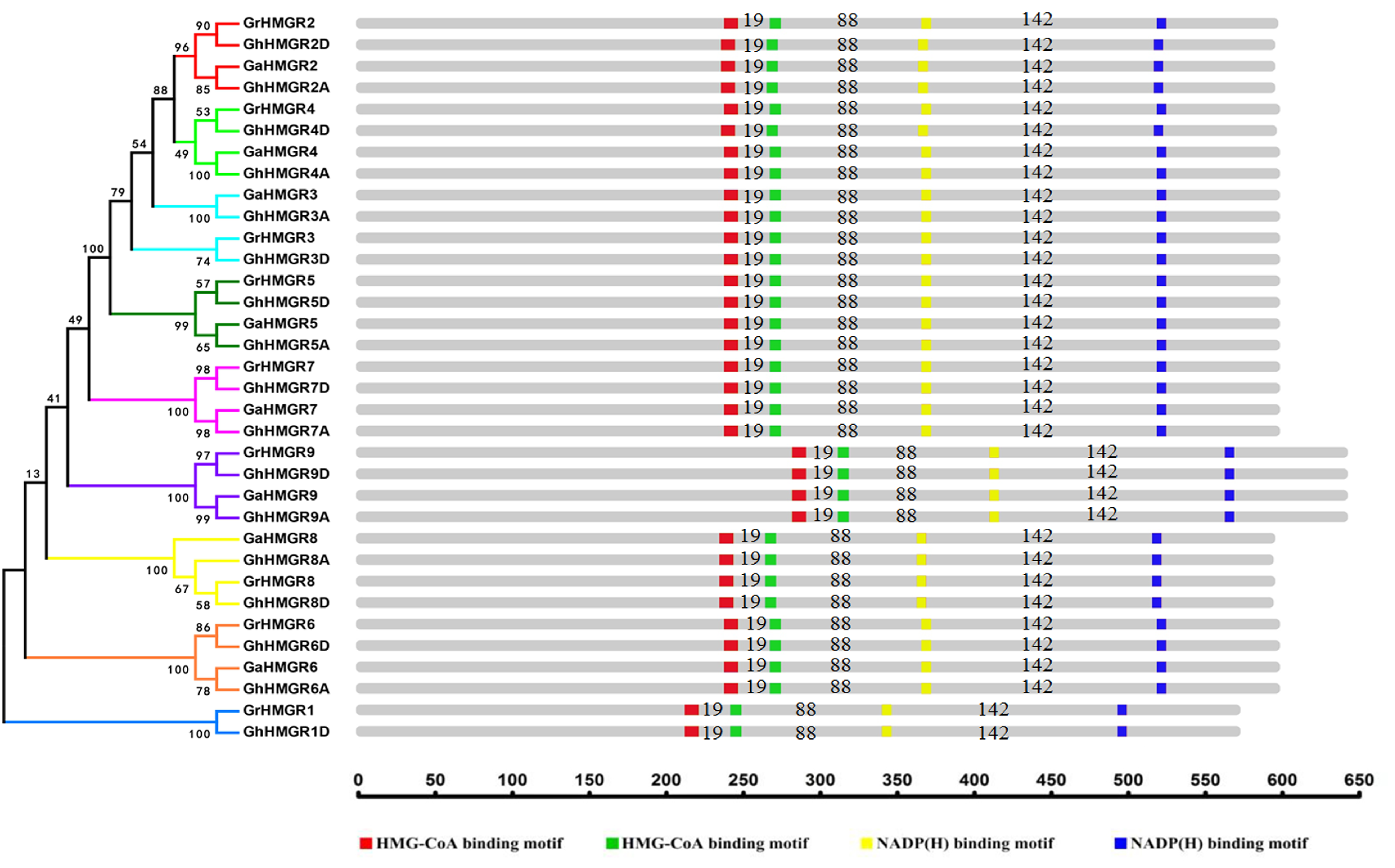

Supplement: Supplementary file 1 [file molecules-23-00193-s001.zip › Supplementary Files/Figure S7.tif]
